# Contact with farming environment as a major risk factor for Shiga toxin (Vero cytotoxin)-producing Escherichia coli O157 infection in humans.

**DOI:** 10.3201/eid0706.010626

**Published:** 2001

**Authors:** S J O'Brien, G K Adak, C Gilham

**Affiliations:** Gastrointestinal Diseases Division, Public Health Laboratory Service Communicable Disease Surveillance Centre, London, England, United Kingdom. sobrien@phls.org.uk

## Abstract

In a prospective, unmatched case-control study of sporadic Shiga toxin (Vero cytotoxin)-producing Escherichia coli O157 (STEC O157) infection in England, exposure to the farming environment emerged strongly as a risk factor (adjusted odds ratio = 2.45; 95% confidence intervals = 1.49-4.02; p=0.0004) posing further challenges and opportunities for prevention.


### Contact with Farming Environment as a Major Risk Factor for Shiga Toxin (Vero Cytotoxin)-Producing *Escherichia coli* O157 Infection in Humans

**Sarah J. O'Brien, Goutam K. Adak, and Clare Gilham**
Public Health Laboratory Service Communicable Disease Surveillance Centre, London, England

In a prospective, unmatched case-control study of sporadic Shiga toxin (Vero cytotoxin)-producing *Escherichia coli* O157 (STEC O157) infection in England, exposure to the farming environment emerged strongly as a risk factor (adjusted odds ratio = 2.45; 95% confidence intervals = 1.49-4.02; p=0.0004) posing further challenges and opportunities for prevention.

Shiga toxin (Vero cytotoxin)-producing *Escherichia coli* O157 (STEC O157) is an important emerging pathogen worldwide, and the illness and death associated with infection are considerable (1). Outbreaks of STEC O157 have been attributed to consuming contaminated food (especially undercooked ground beef) and water, animal contact, and person-to-person transmission (2,3). However, sporadic infection accounts for approximately 80% of all STEC O157 diagnosed in England and Wales (3). Therefore, the sources of and routes of transmission for most infections remain largely unknown. We report the results of a prospective unmatched case-control study, undertaken in England from October 1996 through December 1997. The aim was to identify risk factors for sporadic STEC O157 infection.

### The Study

A patient was defined as a person with abdominal pain or diarrhea (three or more loose stools in a 24-hour period) from whom STEC O157 had been isolated by fecal culture at any of the 47 Public Health Laboratory Service (PHLS) laboratories in England. Patients were included if they were the index patient in the household, normally resided in England, had not traveled abroad in the 5 days before the onset of symptoms, were not part of a known outbreak, and had no evidence of mixed infection. The study took place from October 1, 1996, through December 31, 1997. Ethical approval was obtained from the PHLS Ethics Committee.

A local study coordinator reported positive laboratory results to a central study coordinator at the PHLS Communicable Disease Surveillance Centre (CDSC), complete with details of each patient's general practitioner (GP). The patients' GPs nominated up to three asymptomatic community controls, selected on the basis of gender and age group, for each patient. A standard, structured questionnaire was posted to each study subject along with a reply-paid evelope. The 15-page questionnaire covered demographic and clinical details and food, water, occupational, recreational, and household exposures in the 5 days before the patient's date of onset. Most items were close-ended questions. Nonresponders were sent a second mailing. The data returned to CDSC were entered onto an Epi-Info database (4) and validated by means of double data entry.

Single-risk variable analysis was undertaken by calculating odds ratios and 95% confidence intervals and by continuity-corrected chi-square tests. Variables associated with illness at the 10% significance level in the single-risk variable analysis were included in a logistic regression model. A 10%, rather than the standard 20%, significance level was used because of the large number of variables considered (n=43). Season (October-March and April-September), age group ($\leq$5 years, 6-19, 20-59, and $\geq$60), and gender were included in the model. Terms were assessed by comparing nested models using likelihood ratio tests. Those not reaching a 10% significance level were subsequently rejected from the model. Analyses were performed by using SAS (SAS Institute Inc., Cary, NC) and GLIM (5).

Data were obtained for 369 patients (response rate = 84%) and 511 controls (response rate = 57%). The male-to-female ratio for patients was 1:1. There were, however, slightly more female controls (55.5%) than males. The age range of patients was 2 months to 84 years. Controls were slightly older than patients (median age 21 years for controls, 17 years for patients). Forty-one percent (150/369) of patients were <10 years of age, and 27% (100/369) were <5 years. Sixty-two percent of patients (228/369) had bloody diarrhea, and 38% (140/369) were admitted to hospital.

The risk of developing STEC O157 infection was strongly associated with contact with the farm environment (Table). This encompassed recreational visits by members of the public to open farms (petting zoos) or staying on farms for their holidays (e.g., in farm cottages), and work-related visits to farms. The last category comprised workers (e.g., electricians, maintenance engineers, delivery drivers) who entered farm premises for work purposes but who did not

Address for correspondence: Sarah J. O'Brien, Consultant Epidemiologist and Head, Gastrointestinal Diseases Division, PHLS Communicable Disease Surveillance Centre, 61 Colindale Avenue, London NW9 5EQ, United Kingdom; fax: 44-20-8200-7868; e-mail: sobrien@phls.org.uk

Table. Risk factors for sporadic cases of Vero cytotoxin-producing *Escherichia coli* O157 infection in England: logistic regression analysis

| Variable[a] | Adjusted odds ratio | 95% CI | p value | No. (%) patients exposed in single-risk variable analysis | No. (%) controls exposed in single-risk variable analysis |
|---|---|---|---|---|---|
| Rare chicken | 5.13 | 1.44, 18.26 | 0.009 | 16 (4.7) | 6 (1.3) |
| Purchasing food from a market stall | 2.93 | 1.22, 7.07 | 0.02 | 25 (7) | 17 (3.6) |
| Watercress | 2.61 | 1.24, 5.47 | 0.01 | 29 (8.9) | 23 (4.9) |
| Farm contact | 2.45 | 1.49, 4.02 | 0.0004 | 87 (23.6) | 62 (12.9) |
| Travel (nights away from home) | 2.23 | 1.35, 3.71 | 0.002 | 100 (27.3) | 53 (11.2) |
| Paddling (wading) | 2.13 | 1.04, 4.35 | 0.04 | 40 (11.2) | 24 (5.1) |
| Peaches | 2.08 | 1.17, 3.72 | 0.01 | 53 (15.8) | 37 (7.9) |
| Drank pasteurized milk | 0.66 | 0.43, 1.01 | 0.06 | 240 (68.2) | 351 (74.4) |
| Bought frozen meat | 0.63 | 0.43, 0.95 | 0.03 | 225 (62.5) | 345 (73.6) |
| Ate butter | 0.56 | 0.38, 0.82 | 0.003 | 175 (48.9) | 278 (59.1) |
| Consumed cream | 0.43 | 0.26, 0.7 | 0.0005 | 61 (16.9) | 133 (28.5) |

[a]This model was adjusted for season, age group, and gender and was based on 607 (80%) of the observations (losses due to missing data for one or more of the explanatory variables).
CI = confidence intervals.

regularly work in the farming environment. With respect to recreational visits, approximately half the patients exposed in the single-risk variable analysis reported touching farm animals. The remainder had simply been exposed to the environment. Farmers who routinely worked with livestock were not found to be at increased risk.

Travel away from home during the exposure period was also associated with increased risk for infection. Of those who had spent nights away from home, most (87%) had traveled elsewhere in the United Kingdom as opposed to staying with friends or relatives locally.

Although eating rare chicken and watercress and purchasing food from a market stall were associated with increased risk for STEC O157 infection, these exposures accounted for a very small proportion of patients in the single-risk variable analysis. Consumption of cream and butter and purchasing frozen meat were inversely associated with risk for STEC O157 infection. Eating ground beef was not associated with infection in this study.

### Conclusions

Contact with the farming environment, which included recreational or occupational visits, was strongly associated with sporadic STEC O157 infection in England. The risk occurred in people not routinely exposed to the farming environment, i.e., members of the public visiting open farms or spending holidays on farms, or people who had recently gone onto a farm for work but who were not regularly employed on farms. In contrast with recreational visits, for the work-related visits we were unable to differentiate between animal contact and simply spending time in the farm environment. Although farmers were not found to be at increased risk for infection with STEC O157, we were unable to determine the risk among farmers' children since the questionnaire sought only occupational details and the address information was insufficient to allow us to determine farming premises with accuracy.

We performed an unmatched prospective case-control study using self-administered questionnaires because this design permits efficient study of large numbers of patients and controls. However, we must consider the sources of bias. Patients were recruited through the PHLS national network. We did not include cases diagnosed in National Health Service (NHS) laboratories in order to reduce the opportunity for selection bias based on diagnostic criteria. Since 1995 it has been PHLS policy for all laboratories in the network to test all diarrheal stools by standard protocols (6). Many non-PHLS laboratories appear to use more selective screening protocols, e.g., testing samples from infants and the elderly and samples containing frankly bloody stools. Including cases from NHS sources, therefore, would have favored the selection of infants, the elderly, or those with more severe symptoms. Although this means of patient recruitment might be considered to limit the representativeness of the study, the fact that most cases of STEC O157 in England were diagnosed by the PHLS during the study mitigates this concern.

Matching was not used, the danger being that the patient and control populations might have been systematically different. However, the recruitment of controls through the patients' GPs ensured that controls were drawn from the same population as the patients. Furthermore, the potential confounders of age and gender were included as variables in the logistic regression analysis.

Direct zoonotic and environmental transmission have emerged as important risk factors for outbreaks of STEC O157 in the United Kingdom in recent years (2,7,8). Our results suggest, however, that for sporadic cases of STEC O157, transmission of infection directly from the farm environment to humans appears to be more important than is generally recognized. This means that the patient history for STEC O157 infection and other potentially zoonotic diseases should routinely include a determination of exposure to farm animals or the farm environment.

Our findings are consistent with previous descriptive studies undertaken in Scotland (9) and the southwest of England (10) and the results from a case-control study in Wales (11). These findings indicate opportunities for prevention. People aware of the risks associated with this exposure are empowered to take simple measures to prevent themselves from becoming infected, such as washing their hands after coming into contact with livestock or farm animal feces.

### Acknowledgments

We thank the following: local PHLS study coordinators, PHLS laboratory staff, and general practitioners for their assistance with recruitment of cases and controls; Henry Smith and the staff of the PHLS Laboratory of Enteric Pathogens for reference microbiology; and Neville Verlander for additional statistical support.

The Department of Health provided funding for this research (project no. 241).

Dr. O'Brien is head of the Gastrointestinal Diseases Division at the Public Health Laboratory Service Communicable Disease Surveillance Centre in London. Her professional interests include the surveillance and epidemiology of gastrointestinal infections, outbreak investigations and prevention strategies.

### References

1. Mead PS, Griffin PM. *Escherichia coli* O157:H7. Lancet 1998; 352:1207-12.
2. Milne LM, Plom A, Strudley I, Pritchard GC, Crooks R, Hall M, et al. *Escherichia coli* O157 incident associated with a farm open to members of the public. Commun Dis Public Health 1999;2:22-6.
3. Subcommittee of the PHLS Advisory Committee on Gastrointestinal Infections. Guidelines for the control of infection with Vero cytotoxin-producing *Escherichia coli* (VTEC). Commun Dis Public Health 2000;3:14-23.
4. Dean AD, Dean JA, Burton JH, Dicker RC. Epi-Info, version 5. Atlanta: Centers for Disease Control and Prevention; 1990.
5. Francis B, Green M, Payne C, editors. The GLIM System release 4 manual. Oxford: Clarendon Press; 1993.
6. Vero cytotoxin-producing *Escherichia coli*: which specimens should be tested? Commun Dis Rep CDR Wkly 1995;5:147.
7. Shukla R, Slack R, George A, Cheasty T, Rowe B, Scutter J. *Escherichia coli* O157 infection associated with a farm visitor centre. Commun Dis Rep CDR Rev 1995;5:R86-90.
8. Crampin M, Willshaw G, Hancock R, Djuretic T, Elstob C, Rouse A, et al. Outbreak of *Escherichia coli* O157 infection associated with a music festival. Eur J Clin Microbiol Infect Dis 1999;18:286-8.
9. Coia JE, Sharp JC, Campbell DM, Curnow J, Ramsay CN. Environmental risk factors for sporadic *Escherichia coli* O157 infection in Scotland: results of a descriptive epidemiology study. J Infect 1998;36:317-21.
10. Trevena WB, Willshaw GA, Cheasty T, Domingue G, Wray C. Transmission of Vero cytotoxin producing *Escherichia coli* O157 infection from farm animals to humans in Cornwall and West Devon. Commun Dis Public Health 1999;2:263-8.
11. Parry SM, Salmon RL, Willshaw GA, Cheasty T. Risk factors for and prevention of sporadic infections with Vero cytotoxin (shiga toxin) producing *Escherichia coli* O157. Lancet 1998;351:1019-22
